# Supplementary material for: Genetic associations between circulating immune cells and periodontitis highlight the prospect of systemic immunoregulation in periodontal care
Source: eLife. 2024 Mar 27;12:RP92895. doi: 10.7554/eLife.92895 (PMC10972561; doi:10.7554/eLife.92895)
Supplement: Supplementary file 2. [file elife-92895-supp2.doc]

Supporting information for

**Genetic associations between circulating immune cells and periodontitis highlight the prospect of systemic immunoregulation in periodontal care**

*Xinjian Ye, Yijing Bai, Mengjun Li, Yuhang Ye, Yitong Chen, Bin Liu, Yuwei Dai, Shan Wang, Weiyi Pan, Zhiyong Wang, Yingying Mao & Qianming Chen*

**STROBE-MR Checklist**

| **Section** | **Item No.** | **Checklist item** | **Page No.** |
| --- | --- | --- | --- |
| **Title and abstract** | 1 | Indicate Mendelian randomization (MR) as the study’s design in the title and/or the abstract if that is a main purpose of the study | We have clarified in the abstract section. |
| **Introduction** | | | |
| Background | 2 | Explain the scientific background and rationale for the reported study. What is the exposure? Is a potential causal relationship between exposure and outcome plausible? Justify why MR is a helpful method to address the study question | Evidence from epidemiology and pathophysiology suggests that there is a link between circulating immune cells and periodontitis. The exposure in question is circulating immune cells. The potential causal relationship between this exposure and periodontitis is plausible due to correlational evidence between elevated level of circulating immune cells and the occurence of periodontitis. Mendelian Randomization (MR) is a useful method to address the study question as it helps to estimate causal relationships while controlling for confounding factors. |
| Objectives | 3 | State specific objectives clearly, including pre-specified causal hypotheses (if any). State that MR is a method that, under specific assumptions, intends to estimate causal effects | The specific objectives of the study are to investigate the causal association between circulating immune cells and the risk of periodontitis. The pre-specified causal hypothesis is that circulating immune cells has a causal association with periodontitis.  The study aims to estimate these causal effects by employing multiple Mendelian Randomization (MR) methods, which under certain assumptions, can provide estimates of causal relationships by using genetic variants as proxies for the exposure of interest. |
| **Methods** | | | |
| Study design and data sources | 4 | Present key elements of the study design early in the article. Consider including a table listing sources of data for all phases of the study. For each data source contributing to the analysis, describe the following: |  |
|  |  | a) Setting: Describe the study design and the underlying population, if possible. Describe the setting, locations, and relevant dates, including periods of recruitment, exposure, follow-up, and data collection, when available. | Our study investigated the impact of circulating immune cells on the risk of periodontitis. GWAS data was obtained from the Blood Cell Consortium (BCC), Sardinian cohort, Gene-Lifestyle Interactions in Dental Endpoints (GLIDE), FinnGen (R9K11),GWAS and Sequencing Consortium of Alcohol and Nicotine use (GSCAN), Meta-Analysis of Glucose and Insulin-related Traits Consortium (MAGIC), and GWAS of the Genetic Investigation of ANthropometric Traits (GIANT). |
|  |  | b) Participants: Give the eligibility criteria, and the sources and methods of selection of participants. Report the sample size, and whether any power or sample size calculations were carried out prior to the main analysis | NA |
|  |  | c) Describe measurement, quality control and selection of genetic variants | Candidates for IVs underwent a thorough set of screening procedures. A complicated criterion was performed to equalize the sample disparities among databases. We initially filtered the p-values of the SNPs, followed by the selection of independent SNPs using the linkage disequilibrium (LD) approach. The F-statistics was used to detect weak instrument bias. MR-Egger regression was used to detect the directional pleiotropy. Reverse MR was applied to detect reverse causality. |
|  |  | d) For each exposure, outcome, and other relevant variables, describe methods of assessment and diagnostic criteria for diseases | Table S1 |
|  |  | e) Provide details of ethics committee approval and participant informed consent, if relevant | NA |
| Assumptions | 5 | Explicitly state the three core IV assumptions for the main analysis (relevance, independence and exclusion restriction) as well assumptions for any additional or sensitivity analysis | Our study was predicated on three fundamental assumptions : (1) The Assumption of Relevance: The IVs must demonstrate robust associations with the exposure variable. (2) The Assumption of Independence: The IVs must not correlate with any confounders that concurrently affect both the exposure and the outcome. (3) The Assumption of Exclusion Restriction: The IVs must affect the outcome solely through their impact on the exposure, without any alternative causal pathways. |
| Statistical methods: main analysis | 6 | Describe statistical methods and statistics used |  |
|  |  | a) Describe how quantitative variables were handled in the analyses (i.e., scale, units, model) | NA |
|  |  | b) Describe how genetic variants were handled in the analyses and, if applicable, how their weights were selected | A rigorous threshold of P < 1×10^-9^ was applied to the database with an abundance of positive SNPs (as in the BCC consortium) to ensure the reliability of IVs. Otherwise, a relatively strict standard of P < 1×10^-6^ was initially adopted (as in the Sardinian cohort), and we would loosen it at P < 5×10^-6^ if less than three SNPs met this threshold (an essential requirement for MR-PRESSO analysis). In addition, SNPs that exhibited a direct association with the outcome would also be deleted to support the third MR assumption. Palindromic and ambiguous SNPs were eliminated throughout the harmonization processes to ensure the reliability and validity of causal inference. In MVMR, we excluded SNPs in the major histocompatibility complex area (MHC, 6p21.31) due to their complexity and confounding effects. |
|  |  | c) Describe the MR estimator (e.g. two-stage least squares, Wald ratio) and related statistics. Detail the included covariates and, in case of two-sample MR, whether the same covariate set was used for adjustment in the two samples | The weighted median, maximum likelihood, MR-Egger, and MR-PRESSO global test were used for sensitivity testing to assess the robustness of the IVW estimates.The MR estimators used in the study include the inverse variance weighted (IVW) method, MR-Egger regression, and MR-PRESSO regression. The MR-Egger regression also assesses directional pleiotropy using its intercept term, and Cochran's Q test evaluates heterogeneity among the IVW estimates. Quality control measures are implemented to ensure the validity of the MR estimators. |
|  |  | d) Explain how missing data were addressed | NA |
|  |  | e) If applicable, indicate how multiple testing was addressed | A Bonferroni-corrected criterion of P < 6.27 × 10^-6^ was adopted to measure statistical significance. |
| Assessment of assumptions | 7 | Describe any methods or prior knowledge used to assess the assumptions or justify their validity | NA |
| Sensitivity analyses and additional analyses | 8 | Describe any sensitivity analyses or additional analyses performed (e.g. comparison of effect estimates from different approaches, independent replication, bias analytic techniques, validation of instruments, simulations) | We utilized summary statistics from publicly available genome-wide association studies (GWAS) to perform both univariable MR (UVMR) and multivariable MR (MVMR) analyses. Furthermore, we replicated the UVMR analysis by excluding potentially pleiotropic SNPs and subsequently conducted subgroup and reverse MR analyses. Additionally, the Bayesian model averaging (MR-BMA) was employed to pinpoint the predominant characteristics with causal signals. Finally, we conducted a transcriptome-wide association study (TWAS) and colocalization analysis to discern potential genes implicated in biological relationships. |
| Software and pre-registration | 9 | a) Name statistical software and package(s), including version and settings used | The statistical analyses were performed using "TwoSampleMR" (version 0.5.7), "MRPRESSO" (version 1.0), "RadialMR" (version 1.1), "mrbma" (version 0.1.0), and "GagnonMR" (version 0.0.0.9) packages in R software (version 4.3.1). |
|  |  | b) State whether the study protocol and details were pre-registered (as well as when and where) | NA |
| **Results** | | | |
| Descriptive data | 10 | a) Report the numbers of individuals at each stage of included studies and reasons for exclusion. Consider use of a flow diagram | Figure 1 |
|  |  | b) Report summary statistics for phenotypic exposure(s), outcome(s), and other relevant variables (e.g. means, SDs, proportions) | Table 1, Table S1 |
|  |  | c) If the data sources include meta-analyses of previous studies, provide the assessments of heterogeneity across these studies | NA |
|  |  | d) For two-sample MR:  i. Provide justification of the similarity of the genetic variant-exposure associations between the exposure and outcome samples  ii. Provide information on the number of individuals who overlap between the exposure and outcome studies | Results-Estimated effects of circulating immune cells on periodontitis risk. |
| Main results | 11 | a) Report the associations between genetic variant and exposure, and between genetic variant and outcome, preferably on an interpretable scale | Results-Estimated effects of circulating immune cells on periodontitis risk. |
|  |  | b) Report MR estimates of the relationship between exposure and outcome, and the measures of uncertainty from the MR analysis, on an interpretable scale, such as odds ratio or relative risk per SD difference | Figure 2-3 |
|  |  | c) If relevant, consider translating estimates of relative risk into absolute risk for a meaningful time period | NA |
|  |  | d) Consider plots to visualize results (e.g. forest plot, scatterplot of associations between genetic variants and outcome versus between genetic variants and exposure) | Figure supplement |
| Assessment of assumptions | 12 | a) Report the assessment of the validity of the assumptions | Results-Estimated effects of circulating immune cells on periodontitis risk. |
|  |  | b) Report any additional statistics (e.g., assessments of heterogeneity across genetic variants, such as I2, Q statistic or E-value) | The MR-Egger regression revealed no evidence of horizontal pleiotropy (p-values for intercept > 0.05). However, significant heterogeneity was detected in two traits (memory B cell and monocyte) (Supplementary File 1 — Table s4), which faded after the removal of outliers (Supplementary File 1 — Table s5, Figure 2—figure supplement 1). |
| Sensitivity analyses and additional analyses | 13 | a) Report any sensitivity analyses to assess the robustness of the main results to violations of the assumptions | SNPs with F-statistics < 10 would be removed based on the first MR assumption and SNPs that exhibited a direct association with the outcome would also be deleted to support the third MR assumption. |
|  |  | b) Report results from other sensitivity analyses or additional analyses | Results-Assessing the independent and prioritized relationships through multivariable Mendelian randomization |
|  |  | c) Report any assessment of direction of causal relationship (e.g., bidirectional MR) | Reverse MR revealed no indication of reverse causality (Supplementary File 1 — Table s10). |
|  |  | d) When relevant, report and compare with estimates from non-MR analyses | NA |
|  |  | e) Consider additional plots to visualize results (e.g., leave-one-out analyses) | Figure supplement |
| **Discussion** | | | |
| Key results | 14 | Summarize key results with reference to study objectives | In conclusion, the present study provides suggestive evidence of the causal associations of genetically predicted circulating neutrophils, Natural Killer T cells, and plasmacytoid Dendritic Cells on the risk of periodontitis. |
| Limitations | 15 | Discuss limitations of the study, taking into account the validity of the IV assumptions, other sources of potential bias, and imprecision. Discuss both direction and magnitude of any potential bias and any efforts to address themOur study's conclusions are constrained by limitations such as | Our study's conclusions are constrained by limitations such as the usage of GWAS databases, the unstable results from the IVW method, variation in sample sizes. Additionally, MR evaluates t may correspond differently to fact. |
| Interpretation | 16 | a) Meaning: Give a cautious overall interpretation of results in the context of their limitations in comparison with other studies | Disscussion-Strengths and limitations in the present study. |
|  |  | b) Mechanism: Discuss underlying biological mechanisms that could drive a potential causal relationship between the investigated exposure and the outcome, and whether the gene-environment equivalence assumption is reasonable. Use causal language carefully, clarifying that IV estimates may provide causal effects only under certain assumptions | Disscussion-Circulating neutrophils play a significant part in periodontitis and inflammatory comorbidities. |
|  |  | c) Clinical relevance: Discuss whether the results have clinical or public policy relevance, and to what extent they inform effect sizes of possible interventions | Our findings may provide an innovative and evidence-based framework for systemic immunomodulation management in periodontal care, which can be valuable for early diagnostics, risk assessment, targeted prevention, and personalized management of periodontitis, especially for patients with systemic susceptibility factors. |
| Generalizability | 17 | Discuss the generalizability of the study results (a) to other populations, (b) across other exposure periods/timings, and (c) across other levels of exposure | We could not evaluate the impact of immune cells on distinct subsets of periodontal illnesses (such as gingival recession and periodontal abscess) or ethnic groups (such as East Asian and African). |
| **Other information** | | | |
| Funding | 18 | Describe sources of funding and the role of funders in the present study and, if applicable, sources of funding for the databases and original study or studies on which the present study is based | This research was supported by the Fundamental Research Funds for the Central Universities (No. 2022FZZX01-33 & 2023QZJH58), the National Major Science and Technology Projects of China (No. 81991500 & 81991502), Zhejiang University Global Partnership Fund (No.188170 & 194452307/004), the Joint Natural Science Foundation of Zhejiang Province (No.LHDMD23H300001). |
| Data and data sharing | 19 | Provide the data used to perform all analyses or report where and how the data can be accessed, and reference these sources in the article. Provide the statistical code needed to reproduce the results in the article, or report whether the code is publicly accessible and if so, where | Data from GWAS can be obtained at reported resources: The Polygenic and Monogenic Basis of Blood Traits and Diseases, <https://www.ebi.ac.uk/gwas.> Complex genetic signatures in immune cells underlie autoimmunity and inform therapy, <https://www.ebi.ac.uk/gwas.> Genome-wide analysis of dental caries and periodontitis combining clinical and self-reported data, <https://data.bris.ac.uk/data/dataset.> FinnGen provides genetic insights from a well-phenotyped isolated population, <https://www.finngen.fi/en.> |
| Conflicts of Interest | 20 | All authors should declare all potential conflicts of interest | The authors declared no potential conflicts of interest with respect to the research, authorship, and/or publication of this article. |

**Reference**

1. Skrivankova VW, Richmond RC, Woolf BAR, et al. Strengthening the Reporting of Observational Studies in Epidemiology using Mendelian Randomisation (STROBE-MR): Explanation and Elaboration. BMJ. 2021;375: n2233.
